# Toxicity of Pine Monoterpenes to Mountain Pine Beetle

**DOI:** 10.1038/s41598-017-08983-y

**Published:** 2017-08-18

**Authors:** Christine C. Chiu, Christopher I. Keeling, Joerg Bohlmann

**Affiliations:** 1 Michael Smith Laboratories, University of British Columbia, 2185 East Mall, Vancouver, B.C. V6T, 1Z4 Canada; 2 Botany Department, University of British Columbia, 6270 University Blvd, Vancouver, B.C. V6T 1Z4 Canada; 3 Laurentian Forestry Centre, Natural Resources Canada, P.O. Box 10380, Stn. Sainte-Foy, 1055 du P.E.P.S., Quebec City, QC G1V 4C7 Canada

## Abstract

The mountain pine beetle (*Dendroctonus ponderosae*; MPB) is an eruptive bark beetle species affecting pine forests of western North America. MPB are exposed to volatile monoterpenes, which are important host defense chemicals. We assessed the toxicity of the ten most abundant monoterpenes of lodgepole pine (*Pinus contorta*), a major host in the current MPB epidemic, against adult MPB from two locations in British Columbia, Canada. Monoterpenes were tested as individual volatiles and included (−)-β-phellandrene, (+)-3-carene, myrcene, terpinolene, and both enantiomers of α-pinene, β-pinene and limonene. Dose-mortality experiments identified (−)-limonene as the most toxic (LC_50_: 32 μL/L), and (−)-α-pinene (LC_50_: 290 μL/L) and terpinolene (LC_50_: >500 μL/L) as the least toxic. MPB body weight had a significant positive effect on the ability to survive most monoterpene volatiles, while sex did not have a significant effect with most monoterpenes. This study helps to quantitatively define the effects of individual monoterpenes towards MPB mortality, which is critical when assessing the variable monoterpene chemical defense profiles of its host species.

## Introduction

The mountain pine beetle (*Dendroctonus ponderosae*; MPB) is an eruptive bark beetle that infests different pine (*Pinus sp*.) species in its native range of western North America. Since the late 1990’s, a continuous MPB outbreak has affected over 25 million hectares of lodgepole pine (*Pinus contorta*) forests^1^. The MPB has crossed the geographic barrier of the Rocky Mountains and is expanding its host range from lodgepole pine, which is dominant west of the Rocky Mountains, into jack pine (*P. banksiana*) east of the Rocky Mountains^2^. To overcome a well-defended host tree, MPB employs aggregation pheromones such as female-released (−)-*trans*-verbenol and male-released *exo*-brevicomin to recruit a critical threshold of conspecifics. MPB also benefits from symbiotic relationships with fungal and bacterial associates^3, 4^.

A major chemical defense system of conifers is the production of oleoresin, which consists primarily of a complex mixture of different volatile monoterpenes, non-volatile diterpenoids, and lesser abundant sesquiterpenes^5–7^. Oleoresin terpenoids are produced constitutively and biosynthesis is induced when trees are exposed to biotic stress^8–10^. The terpenoid profiles of the oleoresin vary substantially across different conifer species and between populations and individuals of the same species^11–13^. These variations may be explained, at least in part, by genomic variations of terpene synthase genes^14, 15^. For example the monoterpene profile of lodgepole pine has higher relative amounts of β-phellandrene and terpinolene compared to jack pine, which has higher relative amounts of (+) and (−)-α-pinene, (+)-3-carene, and (−)-limonene^16, 17^.

Coevolution of MPB with conifer monoterpenes resulted in complex chemo-ecological interactions^18, 19^. While pines produce monoterpenes as part of a chemical defense system, MPB can exploit pine monoterpenes as signals for identification of a suitable host, and also incorporates certain monoterpenes to produce pheromones for mass attack to overcome the host defense. For example, (−)-α-pinene serves as the precursor to (−)-*trans*-verbenol^20^, an aggregation pheromone produced and released by females upon initial attack of a host tree. Female MPBs produce *trans*-verbenol after phloem feeding^21–23^ and after exposure to α-pinene vapours at concentrations of 25–250 μL/L^24–26^. In addition, the host monoterpenes terpinolene and myrcene are synergists of aggregation pheromones as the addition of these monoterpenes to pheromone baits increases the number of attracted beetles compared to the pheromone alone^27–29^.

There is considerable evidence that monoterpenes are toxic to insects. For example, dose-response studies of monoterpenes with cockroach (*Blattella germanica*)^30^, flour beetle (*Tribolium confusum*)^31^, cabbage looper (*Trichoplusia ni*)^32^, and housefly (*Musca domestica*)^33^, using fumigant and contact exposure, found that monoterpenes are toxic at doses comparable to those of synthetic pesticides. The mode of action of monoterpenes at the molecular level appears to include components of the insect nervous system, specifically octapamine and tyramine receptors^34^, acetylcholinesterase^35, 36^ and GABA receptors^37^. Toxicity studies of saturated pine resin vapours with *Dendroctonus* species, specifically the western pine beetle (*D. brevicomis*), Jeffery pine beetle (*D. jeffreyi*), and mountain pine beetle (*D. ponderosae*), found that non-host pine resin is more toxic that host pine resin^38–40^. This indicates that there are differences in the toxicities of the compounds that comprise the volatile pine profiles. Studies with the western pine beetle (*D. brevicomis*)^41^, the spruce beetle (*D. rufipennis*)^42^, the larch beetle (*D. simplex*)^42^, and southern pine beetle (*D. frontalis*)^43^ showed that limonene is more toxic when compared to other monoterpenes (typically α-pinene, β-pinene, 3-carene and myrcene). For MPB however, the toxicity of the individual monoterpenes that dominate the volatile profiles of its hosts is not known.

Here, we quantitatively tested the toxicity of individual monoterpene volatiles that are abundant in lodgepole pine using dose-mortality experiments with beetles sourced from two locations in British Columbia, Canada. In line with previous work and to enable comparison with the literature, we used exposure to monoterpene volatiles, as opposed to contact exposure. Results are reported as LC_50_, which is the concentration at which 50% of a MPB test population is killed by a given substance after 24 h, and thereby provides a benchmark for comparison between different monoterpenes. MPB body weight varies by over three-fold, and females, the pioneering sex, are on average heavier than males. Thus, body weight and sex were monitored to assess their influence on beetle survival with different monoterpenes.

## Results

### LC_50_ varies by monoterpene with (−)-limonene being the most toxic against MPB

We measured the LC_50_ values for ten different monoterpenes at 24 h of vapour exposure with MPB from two locations, cohorts 1 and 2 (Tables 1 and 2). We analyzed the cohorts separately because the beetles from these two cohorts differed significantly in weight (two sample t-test, p < 0.0001). The LC_50_ values ranged from ~30 μL/L to >500 μL/L, revealing a quantitative difference in toxicity of over ten-fold between monoterpenes. Overall, the results were consistent with beetles from the two cohorts with respect to the relative toxicity of most of the monoterpenes tested. The most toxic monoterpene was (−)-limonene, which was significantly more toxic than the (+)-limonene enantiomer and any of the other monoterpenes. The next most toxic terpenes were (+)-3-carene, myrcene, and (−)-β-phellandrene, followed by the two enantiomers of β-pinene, which were close in LC_50_ value, and (+)-α-pinene. Notably, (−)-α-pinene was considerably less toxic than the (+) enantiomer, and this difference was significant in MPB from cohort 2. The LC_50_ value for terpinolene could not be determined definitively, because too few of the beetles died even at the highest concentrations, revealing that terpinolene was much less toxic than any of the other monoterpenes tested.

**Table 1:** Toxicity of monoterpene volatiles against MPB from cohort 1.

Cohort 1					
Monoterpenes	N	LC_50_ [μl/L] (f.l.)	Hill Slope ± SE	χ^2^ (df)	Mean Weight [mg] ± SE
(−)-Limonene	144	49 (34–71)^a^	−1.6 ± 0.3	5.4 (3)	9.33 ± 0.22
(+)-Limonene	120	89 (66–120)^b^	−2.1 ± 0.6	8.5 (1)	8.51 ± 0.19
(+)−3-Carene	120	117 (86–158)^bc^	−2.3 ± 0.6	8.8 (3)	8.66 ± 0.19
Myrcene	119	163 (113–234)^cd^	−2.2 ± 0.6	4.0 (3)	8.54 ± 0.21
(+)-α-Pinene	120	185 (151–227)^cd^	−4.1 ± 1.2	4.6 (3)	8.63 ± 0.19
(−)-β-Pinene	120	221 (164–296)^d^	−3.3 ± 1.2	2.3 (3)	8.94 ± 0.19
(−)-α-Pinene	120	277 (160–477)^d^	−1.4 ± 0.4	5.3 (3)	9.07 ± 0.20
Terpinolene	120	>500			8.74 ± 0.21

Dose-response models of the logit-transformed mortality data were used to determine the LC_50_ values, 95% fiducial limits (f.l.), and Hill slope for each monoterpene tested. LC_50_ values denoted with the same letter were not significantly different. Multiple comparisons between LC_50_ values were conducted via pairwise t-tests on the log (LC_50_) values of the Hill equation, using a pooled standard error, and correcting for experiment-wise error by the Benjamini–Hochberg procedure. The goodness-of-fit test χ^2^ (degree of freedom) was used to assess the fit of the dose-response model. Less than 50% of the beetles died at all doses of terpinolene. The mean weight of the beetles tested for each compound in cohort 2 was not significantly different and the mean weight of the beetles tested with a given monoterpene was the same at all doses (ANOVA, p-value = not significant). As sex was not a significant factor in mortality for all monoterpenes tested, the data for both sexes was combined.

**Table 2:** Toxicity of monoterpene volatiles against MPB from cohort 2.

Cohort 2					
Monoterpenes	N	LC_50_ [μl/L] (f.l.)	Hill slope ± SE	χ^2^ (df)	Mean Weight [mg] ± SD
(−)-Limonene	120	32 (22–47)^a^	−2.2 ± 0.5	1.4 (2)	10.03 ± 0.19
(+)-Limonene	120	60 (52–70)^b^	−5.9 ± 1.6	<0.1 (1)	9.68 ± 0.23
(+)−3-Carene	120	76 (63–90)^bc^	−4.5 ± 1.0	0.4 (1)	9.47 ± 0.19
Myrcene	119	77 (55–108)^bc^	−1.6 ± 0.3	1.5 (2)	9.76 ± 0.22
(−)-β-Phellandrene	120	97 (75–124)^cd^	−3.0 ± 0.7	9.1 (3)	9.52 ± 0.22
(−)-β-Pinene	120	130 (105–160)^d^	−4.9 ± 1.4	<0.1 (1)	9.52 ± 0.18
(+)-β-Pinene	120	131 (108–158)^d^	−6.7 ± 1.9	0.6 (3)	9.45 ± 0.20
(+)-α-Pinene	120	131 (106–161)^d^	−5.0 ± 1.4	3.5 (3)	9.99 ± 0.19
(−)-α-Pinene	120	248 (187–330)^e^	−2.2 ± 0.5	0.6 (1)	9.73 ± 0.21
Terpinolene	120	>500			9.73 ± 0.21

Dose-response models of the logit-transformed mortality data were used to determine the LC_50_ values, 95% fiducial limits (f.l.), and Hill slope for each monoterpene tested. LC_50_ values denoted with the same letter were not significantly different. Multiple comparisons between LC_50_ values were conducted via pairwise t-tests on the log (LC_50_) values of the Hill equation, using a pooled standard error, and correcting for experiment-wise error by the Benjamini–Hochberg procedure. The goodness-of-fit test χ^2^ (degree of freedom) was used to assess the fit of the dose-response model. Less than 50% of the beetles died at all doses of terpinolene. The mean weight of the beetles tested for each compound in cohort 2 was not significantly different and the mean weight of the beetles tested with a given monoterpene was the same at all doses (ANOVA, p-value = not significant). As sex was not a significant factor in mortality for all monoterpenes tested, the data for both sexes was combined.

### Factors affecting mortality

Logistical regression analyses (Tables 3 and 4; Supporting Figures S1 and S2) showed that monoterpene concentration had a significant positive relationship with mortality for all monoterpenes tested. Body weight negatively influenced mortality, i.e. heavier MPB showed increased survival with most monoterpenes. MPB from the two cohorts differed significantly in average weight (two sample t-test, p < 0.0001). The body weight (average fresh weight ± SD) of MPB was 8.8 mg ± 2.2 mg for cohort 1 (N = 983) and 9.7 mg ± 2.2 mg for cohort 2 (N = 1199) (Supporting Figures S3 and S4). For both locations, increased body weight correlated significantly with higher survival in the treatments with (−)-limonene, myrcene, (−)-β-pinene and terpinolene (Tables 3 and 4). The monoterpene (+)-β-pinene also showed this relationship, but was only tested with beetles from cohort 2. MPB with higher body weight also survived treatment with (+)-α-pinene significantly more often in cohort 1, but this relationship was not found in cohort 2, and was the opposite with (−)-α-pinene. Body weight did not affect the survival in treatments with (+)-limonene or (−)-β-phellandrene. Sex did not have a significant effect on mortality with most monoterpenes. However, males from cohort 1 survived better than females in treatments with (+)-3-carene and (−)-β-pinene, but this relationship was not found with MPB of the cohort 2.

**Table 3:** The independent effects of concentration, body weight and sex on mortality for each monoterpene tested with MPB from cohort 1.

Cohort 1				
Monoterpene		Concentration	Weight	Sex
(−)-Limonene	Coefficient (±S.E.)	0.018 ± 0.004	−0.38 ± 0.12	−0.05 ± 0.48
	*P*	8.75e-07*	0.001*	0.915
(+)-Limonene	Coefficient (±S.E.)	0.018 ± 0.004	−0.23 ± 0.15	0.52 ± 0.59
	*P*	1.21e-06*	0.115	0.379
(+)-3-Carene	Coefficient (±S.E.)	0.016 ± 0.003	−0.54 ± 0.17	−1.22 ± 0.59
	*P*	5.77e-06*	0.002*	0.039*
Myrcene	Coefficient (±S.E.)	0.012 ± 0.003	−0.40 ± 0.14	0.78 ± 0.54
	*P*	2.95e-06*	0.005*	0.148
(+)-α-Pinene	Coefficient (±S.E.)	0.019 ± 0.004	−0.46 ± 0.21	0.30 ± 0.73
	*P*	5.61e-06*	0.030*	0.680
(−)-β-Pinene	Coefficient (±S.E.)	0.013 ± 0.003	−0.84 ± 0.23	−1.35 ± 0.67
	*P*	9.79e-07*	<0.001*	0.046*
(−)-α-Pinene	Coefficient (±S.E.)	0.005 ± 0.001	−0.10 ± 0.11	0.26 ± 0.47
	*P*	5.11e-05*	0.360	0.579
Terpinolene	Coefficient (±S.E.)	0.004 ± 0.001	−0.52 ± 0.17	0.26 ± 0.53
	*P*	0.00436*	0.002*	0.625

Logistic regression analysis was used to assess the effects of monoterpene concentration, body weight, and sex on mortality for each of the monoterpenes tested. The coefficient indicates the magnitude and direction of each of these effects on mortality. Concentration: A positive coefficient indicates a positive relationship between increasing concentration and mortality. A significant positive coefficient was found for all monoterpenes tested indicating increasing toxicity at increasing concentrations. Weight: A positive coefficient indicates a positive relationship between increasing weight and mortality. Most monoterpenes had a significant negative coefficient, indicating heavier beetles survived more often. Sex: A positive coefficient indicates females survived more often than males. A negative coefficient indicates males survived more often than females. Most monoterpenes did not show a significant coefficient indicating that sex did not influence survival. *Denotes *P*-value < 0.05.

**Table 4:** The independent effects of concentration, body weight and sex on mortality for each monoterpene tested with MPB from cohort 2.

Cohort 2				
Monoterpene		Concentration	Weight	Sex
(−)-Limonene	Coefficient (±S.E.)	0.084 ± 0.019	−0.66 ± 0.27	−1.14 ± 0.92
	*P*	8.3e-06*	0.017*	0.215
(+)-Limonene	Coefficient (±S.E.)	0.064 ± 0.013	−0.25 ± 0.17	−0.51 ± 0.83
	*P*	1.77e-06*	0.147	0.540
(+)-3-Carene	Coefficient (±S.E.)	0.051 ± 0.011	−0.42 ± 0.22	0.36 ± 0.78
	*P*	1.76e-06*	0.059	0.650
Myrcene	Coefficient (±S.E.)	0.030 ± 0.006	−0.60 ± 0.17	0.67 ± 0.63
	*P*	9.74e-07*	<0.001*	0.290
(−)-β-Phellandrene	Coefficient (±S.E.)	0.020 ± 0.004	−0.18 ± 0.12	−0.14 ± 0.57
	*P*	9.42e-07*	0.158	0.805
(−)-β-Pinene	Coefficient (±S.E.)	0.028 ± 0.005	−0.58 ± 0.21	−0.28 ± 0.70
	*P*	3.6e-07*	0.005*	0.686
(+)-β-Pinene	Coefficient (±S.E.)	0.035 ± 0.007	−0.65 ± 0.23	−1.13 ± 0.86
	*P*	7.7e-07*	0.006*	0.187
(+)-α-Pinene	Coefficient (±S.E.)	0.022 ± 0.004	0.05 ± 0.15	0.70 ± 0.61
	*P*	2.15e-07*	0.733	0.254
(−)-α-Pinene	Coefficient (±S.E.)	0.012 ± 0.002	−0.40 ± 0.16	0.72 ± 0.71
	*P*	1.08e-07*	0.014*	0.313
Terpinolene	Coefficient (±S.E.)	0.005 ± 0.001	−0.37 ± 0.12	0.44 ± 0.50
	*P*	0.000413*	0.002*	0.375

Logistic regression analysis was used to assess the effects of monoterpene concentration, body weight, and sex on mortality for each of the monoterpenes tested. The coefficient indicates the magnitude and direction of each of these effects on mortality. Concentration: A positive coefficient indicates a positive relationship between increasing concentration and mortality. A significant positive coefficient was found for all monoterpenes tested indicating increasing toxicity at increasing concentrations. Weight: A positive coefficient indicates a positive relationship between increasing weight and mortality. Most monoterpenes had a significant negative coefficient, indicating heavier beetles survived more often. Sex: A positive coefficient indicates females survived more often than males. A negative coefficient indicates males survived more often than females. All monoterpenes did not show a significant coefficient indicating that sex did not influence survival. *Denotes *P*-value < 0.05.

## Discussion

MPB are exposed to monoterpenes as volatiles as well as through contact and feeding. For the purpose of this study to assess and compare toxicity of individual monoterpenes, we focused on the exposure to monoterpene volatiles. The effects that monoterpenes have on MPB through physical contact or ingestion were not tested in this study. It is also important to note that there are limitations in the comparison of the LC_50_ values reported here, which are in units of relative volumes, with the absolute amounts of monoterpenes in host trees, which are typically determined after solvent extraction and reported as μg of monoterpene per g of dry weight of tissue sample. Nevertheless, it is reasonable to compare the relative ranking of toxicity of the different individual monoterpenes against MPB described here with the reported relative composition of monoterpene profiles of the host pines. The three most abundant compounds of the monoterpene profile of lodgepole pine are (−)-β-phellandrene (>50%), (−)-β-pinene (up to 35%), and (+)-3-carene (up to 10%)^16, 17^. In another host, ponderosa pine (*Pinus ponderosa*), the three most abundant monoterpenes are (+)-3-carene, β-pinene, and myrcene^44^. In whitebark pine (*Pinus albiculus*), an occassional host of MB, the three most abundant monoterpenes are (−)-β-phellandrene, (−)-β-pinene, and myrcene^12^. These monoterpenes that are relatively abundant in lodgepole pine, ponderosa pine and whitebark pine are of only mid-range toxicity to MPB compared to the other monoterpenes present (Table 1 and 2). Conversely, the two most toxic monoterpenes, the two enantiomers of limonene, account for only 1–5% of the monoterpene profile of lodgepole pine and an even lower percentage of the whitebark pine and ponderosa pine profile^12, 16, 17, 44^. In other bark beetle species, both enantiomers of limonene were also more toxic than other monoterpenes. For example, (+)-limonene volatiles had a shorter LT_50_ (lethal time) compared to other monoterpenes in the spruce beetle (*D. rufipennis*) and the larch beetle (*D. simplex*)^42^, and the exposure of 50 μL/L of limonene to westen pine beetle (*D. brevicomis*) for four days caused more mortality than other monoterpenes^41^. (−)-α-Pinene, the precursor to the aggregation pheromone (−)-*trans*-verbenol^20^, was one of the least toxic monoterpenes to MPB. (+)-α-Pinene, which was significantly more toxic than (−)-α-pinene, is the precursor to (+)-*trans*-verbenol, a compound that is also produced by female MPB, but has not been shown to be attractive to beetles^45^.

The most abundant monoterpenes in the co-evolved lodgepole pine and ponderosae pine host were not the most toxic to MPB, and the most toxic monoterpenes, i.e. the limonenes, are not the most abundant. The most abundant monoterpenes in jack pine, a host that does not share the same co-evolutionary association with MPB, are (+)-α-pinene (up to 40%), (+)-3-carene (up to 20%), and (−)-β-pinene (up to 18%)^16, 17^, and these were also of mid- to low-range toxicity against MPB. Like lodgepole pine, the two enantiomers of limonene account for only a small proportion (1–8%) of the jack pine phloem. Based on these comparisons, it is not obvious if the monoterpene defense of jack pine will be more or less effective against MPB adults than that of lodgepole pine. This is consistent with studies in other bark beetle systems showing that minor monoterpene components were of either greater or equal toxicity than the major components. For example, the toxicity of jack pine and red pine components to *Ips pini* ^46^, loblolly pine and other southern pine components to *D. frontalis* ^43^, and grand fir components to *Scolytus ventalis* ^47^.

Boone *et al*.^10^ reported the profiles of constitutive and induced monoterpenes during the endemic, incipient and epidemic population phases of MPB in lodgepole pine. Trees that were subsequently attacked by MPB during the endemic and incipient phases had higher proportions of α-pinene and β-pinene in the constitutive resin, while the proportions of these compounds were lower in trees that were attacked during the epidemic phase. Similar to the induced defenses, the amount of β-pinene was also higher in trees that were attacked during endemic and incipient phases^10^. The relative abundance of the less toxic α-pinene and β-pinene could make trees an easier target at lower MPB population densities. Myrcene, a monoterpene of mid-range toxicity to MPB, was more highly induced in trees that were attacked in the epidemic phase^10^. Limonene, which appears to be the most toxic monoterpene against MPB, was also the most strongly induced monoterpene terpene^10^; however, it did not explain the likelihood of a tree being attacked at any of the MPB population phases^10^. In another study, lodgepole pines with higher induced levels of limonene, but lower constitutive levels limonene, were more likely to survive MPB attack^48^.

The diversity and variability of host monoterpene profiles is determined by the multi-member monoterpene synthase (mono-TPS) gene family and the expression of these genes, which differs between conifer species and between individuals of the same species^14^. Within a given tree, monoterpene profiles can change as a result of induced changes of mono-TPS gene and enzyme expression in response to insect attack^9, 49^. Many of the conifer mono-TPSs also produce multiple monoterpenes and the specific profiles of such multi-product mono-TPSs can vary even between very closely related orthologous or paralogous mono-TPSs^15^ adding to the overall complexity and variability of conifer monoterpene profiles. Several mono-TPSs of lodgepole pine and jack pine have been functionally characterized^16^. Most of the monoterpenes in the these two species are produced by more than one mono-TPS. For example, (−)-β-phellandrene, the most abundant monoterpene in lodgepole is the major product of two mono-TPSs, PcTPS-(−)-β-phell1 and PcTPS-(−)-β-phell2. (−)-β-pinene is a major product of PcTPS-(−)-β-pin1 but a minor product of PcTPS-(−)-α-pin1 and PcTPS-(−)-camp/(+)-α-pin1. To date, no mono-TPS that produces either enantiomer of limonene has been functionally characterized from lodgepole pine. However, one mono-TPS, PbTPS-αterp from jack pine produces (−)-limonene as a minor component (5%) of the product profile of this enzyme^16^. Thus, it is currently not known which mono-TPS gene in either of these two host species of MPB is responsible for the formation of the most toxic monoterpene volatile.

Given the evolutionary and life cycle context of MPB exposure to monoterpenes, it is reasonable to hypothesize that MPB has a higher tolerance to monoterpenes than insects that are not typically experiencing high concentrations of monoterpenes in their environment. Indeed, volatile (+)-limonene has an LC_50_ of 5 μL/L in the red flour beetle (*Tribolium casteneum*), and a KD_50_ (knockdown dose) of 7.5 μL/L in the housefly (*Musca domestica*)^31, 33^. By comparison, we showed a higher tolerance of MPB to (+)-limonene with LC_50_ of 60–89 μL/L. The relatively high tolerance of MPB to monoterpene volatiles may be characteristic of coniferophagous bark beetles. For example, the great spruce bark beetle (*Dendroctonus micans*) and the European spruce bark beetle (*Ips typographus*) appeared to be unaffected or had low mortality when exposed to saturated vapours of α-pinene, β-pinene, (+)-3-carene, limonene, and myrcene^50^.

Tolerance of insects to insecticides or plant defense compounds often increases in proportion to body weight^51^. We found a significant relationship between MPB survival and body weight for most monoterpenes. This is in agreement with findings of Reid and Purcell^52^, who showed that the body condition index (a metric calculated from the body weight) explained survival of MPB to high concentrations of (−)-α-pinene, myrcene, (+)-limonene and terpinolene vapours. After accounting for body weight, sex did not have a significant effect on the survival with most of the monoterpenes. This is also in agreement with the findings of Reid and Purcell^52^, despite female MPB being the first to attack a host tree. In a recent study, Reid *et al*.^53^ also tested the effect of four different monoterpenes on MPB mortality in the context of beetle size and vigor. Their results pointed to beetle conditions and terpene concentrations as major factors affecting beetle mortality. However, the small number of only four different monoterpenes tested by Reid *et al*.^53^ is not representative of the true structural diversity of the terpenes of pine oleoresin^16^, and conclusions about the effect of terpene structural diversity may have to be considered with caution.

Pierce *et al*.^22^ explored possible routes of detoxification for monoterpenes and identified oxidized metabolites of monoterpenes in extracts of female MPBs that had fed on pine phloem. We recently showed that the MPB cytochrome P450 enzyme CYP345E2 hydroxylates or epoxidizes α-pinene, β-pinene, (+)-3-carene, limonene and terpinolene, but is found only in the antennae and likely plays an olfaction-specific role, rather than a detoxification role^54^. Genome and transcriptome annotation of the MPB revealed 85 different P450 genes^55, 56^. The MPB P450 gene family showed blooms in the CYP6 and CYP9 clades. Members of these clades have been shown in other insects to be involved in the detoxification of plant host defense compounds and insecticides^57^. Robert *et al*.^58^ found several CYP6 and CYP9 members up-regulated in MPB after feeding on host phloem. In *Ips pini*, a member of the CYP9 family, CYP9T2 hydroxylates myrcene to ipsdienol, a pheromone of this species^59^; and in a related bark beetle, *Ips paraconfusus*, CYP9T1 is up-regulated nearly five orders of magnitude in males following feeding on monoterpene-laden pine phloem^60^.

Beyond directly affecting MPB, monoterpenes may also affect other components and interactions of the symbiotic complex of MPB and its fungal and bacterial microbiomes. For example, the MPB fungal associate *Grosmannia clavigera* utilizes (+)-limonene as a sole carbon source and uses a unique ABC transporter for controlling levels of this monoterpene^61, 62^. Thus, (+)-limonene, which is highly toxic to MPB, may be better tolerated by the fungal associate. Bacterial associates of the MPB also appear to tolerate limonene, with bacterial isolates from MPB growing, or growth being stimulated, by the presence of 1–5% limonene in culture^4^. A metagenomic analysis of MPB associated species of the *Serratia*, *Pseudomonas*, *Burkholderia*, and *Rahnella* genera revealed several genes annotated with possible functions in limonene degradation^63^.

## Methods

### Insects

Beetles were obtained from two locations in British Columbia (BC), Canada. Cohort 1 was from a naturally infested lodgepole pine near Mt Baldy, BC (49°06′32.8″N 119°14′48.1″W), which was felled and the logs collected in May 2015. Cohort 2 was from near Whistler, BC (50°12′33.3″N 122°53′05.2″W), where MPB pheromone baits (Contech) were attached to two lodgepole pines on June 1st 2015 and were removed after signs of attack were apparent. The attacked trees were felled in Oct 2015. Infested logs were placed in screened cages at room temperature to rear beetles to maturity. Emerged beetles were collected every 2 to 4 days and held on moist Kimwipe paper (Kimberly-Clark) at 4 °C until use. Beetles were sexed based on the dimorphism of the seventh abdominal tergite^64^. Beetles that responded weakly to being picked up with forceps during this step were not used in the assay. Each beetle was weighed to the nearest 0.1 mg on the day of use in toxicity assays. Beetles were between 3 and 21 days post-emergence at the time of experiments.

### Monoterpene toxicity assays

Ten monoterpenes (Supplementary Table S1) were used individually for toxicity assays. Monoterpenes were selected based on relative content of 2% or higher in the monoterpene profile of lodgepole pine according to two recent reports^16, 17^. They also included nine of the ten most abundant monoterpenes in jack pine. A 1.5 cm × 1.5 cm piece of Whatman filter paper was placed in a 20-mL scintillation vial (VWR). Defined volumes (see below) of undiluted monoterpenes, monoterpenes diluted into acetone, or acetone as a control were applied to the filter paper using a microdispenser (VWR) immediately before a single beetle was added to the vial and the vial was sealed. Monoterpenes were tested at five defined doses (volume monoterpene applied/volume airspace of the assay vial) of 10 μL/L, 50 μL/L, 100 μL/L, 200 μL/L and 500 μL/L. To achieve the doses of 50 μL/L, 100 μL/L, 200 μL/L and 500 μL/L undiluted monoterpenes were applied at volumes of 1 μL, 2 μL, 4 μL, and 10 μL, respectively. For the 10 μL/L dose, monoterpenes were diluted five-fold in acetone and 1 μL of the diluted monoterpene applied. To control for solvent effects, and as the 0 μL/L control, 1 μL of acetone was applied. MPB were exposed to volatiles for 24 hours and then removed from assay vials to assess mortality. MPB were considered dead if they did not respond after being tapped with soft forceps. In total approximately 120 MPB were tested for each of the two cohorts and with each of the monoterpenes. At each concentration plus control for each monoterpene, 20 MPB with 10 females and 10 males were used. MPB were distributed so that their average body weight was the same for all concentrations in a given cohort trial. Two trials were done on each monoterpene, one with MPB from cohort 1 and the other from cohort 2, except assays with (+)-β-pinene and (−)-β-phellandrene could not be completed with cohort 1 due to insufficient numbers of MPB collected from this location.

### Statistical Analyses

Analyses were conducted using the statistical program language R version 3.4.0^65^. The average beetle weights differed significantly between cohorts (two-tailed t-test, t(2.097) = −9.0627, *p*-value < 0.0001, see also Figures S3 and S4). We therefore analyzed each cohort separately. The drc_3.0–1 package^66^ was used to subject mortality data to logit analysis^67^ using the Hill three-parameter log-logistic function (LL2.3 u(upper = 1)) from which the LC_50_, 95% fiducial limits, Hill slope (b ± S.E.), and χ^2^ values for each compound separately were generated. Multiple comparisons between LC_50_ values were conducted via pairwise t-tests on the log(LC_50_) values of the Hill equation, using a pooled standard error, and correcting for experiment-wise error by the Benjamini–Hochberg procedure. Logistical regression was used for each compound separately to assess the independent effects of concentration, sex and weight on survival.

## Electronic supplementary material

Supplementary Information
