# Supplementary material for: Toxicity of Pine Monoterpenes to Mountain Pine Beetle
Source: Sci Rep. 2017 Aug 18;7:8858. doi: 10.1038/s41598-017-08983-y (PMC5562797; doi:10.1038/s41598-017-08983-y)
Supplement: Supplementary file 1 — Supplementary Information [file 41598_2017_8983_MOESM1_ESM.pdf]

SUPPLEMENTARY INFORMATION

Toxicity of Pine Monoterpenes to Mountain Pine Beetle

Christine C. Chiu[1,2], Christopher I. Keeling[1,3], Joerg Bohlmann[1,2,*]

[1]Michael Smith Laboratories, University of British Columbia, 2185 East Mall, Vancouver, B.C., Canada, V6T 1Z4

[2]Botany Department, University of British Columbia, 6270 University Blvd, Vancouver, B.C., V6T 1Z4

[3]Present address: Department of Biological Sciences, Simon Fraser University, 8888 University Drive, Burnaby, B.C., Canada, V5A 1S6

*corresponding author: E-mail: bohlmann@msl.ubc.ca

Table S1. Monoterpenes used in toxicity bioassays

Compound	CAS	Purity	Supplier	Catalog #	Lot #
(1*R*)-(+)-α-Pinene	7785-70-8	98%	Sigma Aldrich	P45680	08926AE
(1*S*)-(-)-α-Pinene	7785-26-4	99%	Sigma Aldrich	274399	09409MS
(1*S*)-(-)-β-Pinene	18172-67-3	99%	Sigma Aldrich	112089	00307LG
(1*R*)-(+)-β-Pinene	19902-08-0	98%	Fluka	80607	BCBK2147V
(1*S*)-(+)-3-Carene	498-15-7	99%	Sigma Aldrich	441619	0531PH
Terpinolene	586-62-9	85%	Fluka	86485	1401137
(1*S*)-(-)-Limonene	5989-54-8	96%	Sigma Aldrich	21836-7	14105EU
(1*R*)-(+)-Limonene	5989-27-5	~90%	Fluka	62122	BCBH9990V
Myrcene	123-35-3	99%	Sigma Aldrich	M100005	BCBD7911V
(-)-β-Phellandrene	555-10-2	84%	Synergy Semiochemicals	Custom	Custom

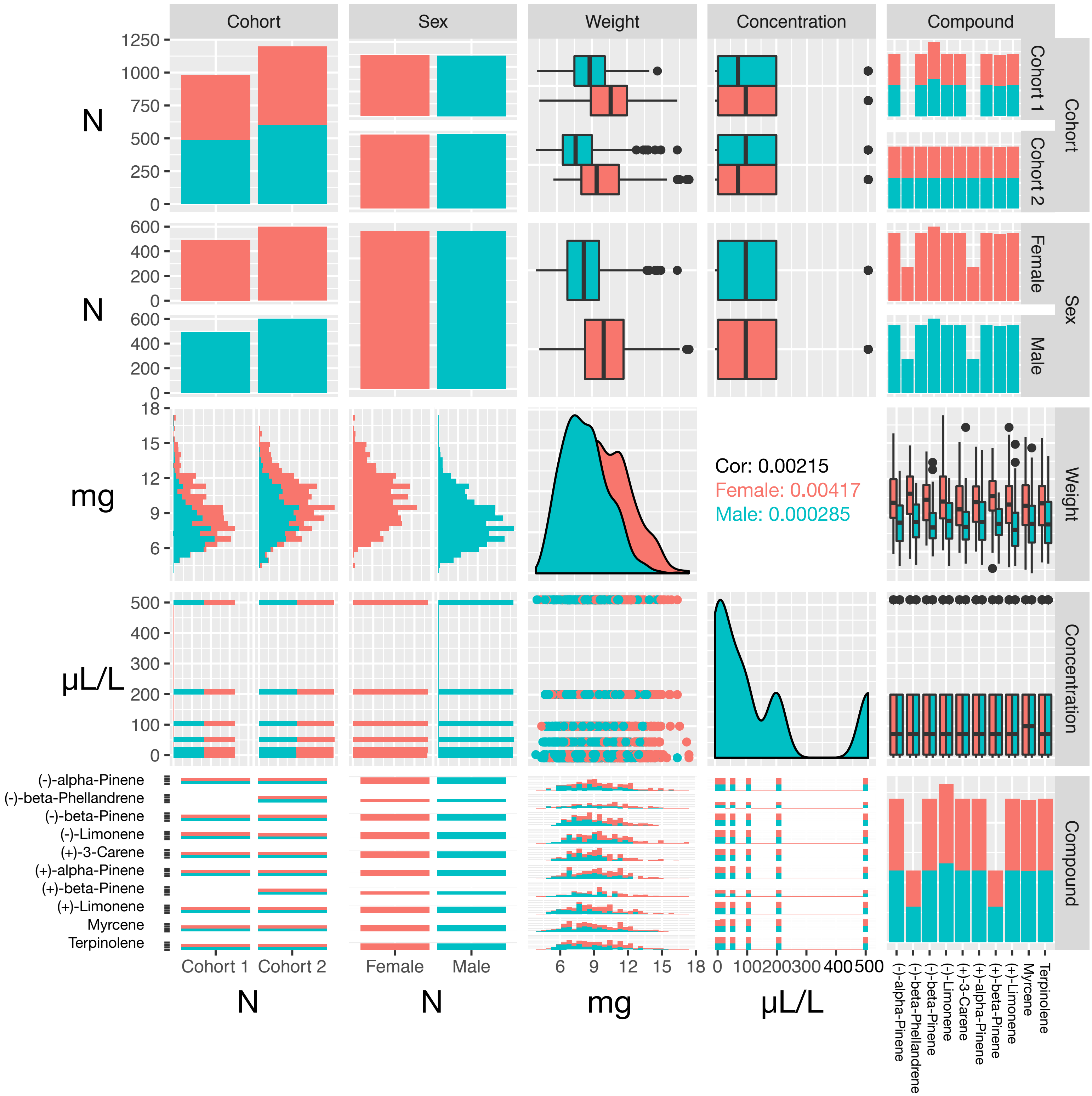

Cohort
Sex
Weight
Concentration
Compound
Cohort 1
Cohort 2
Female
Male
Cor: 0.00215
Female: 0.00417
Male: 0.000285
N
mg
µL/L
(-)-alpha-Pinene
(-)-beta-Phellandrene
(-)-beta-Pinene
(-)-Limonene
(+)-3-Carene
(+)-alpha-Pinene
(+)-beta-Pinene
(+)-Limonene
Myrcene
Terpinolene

Figure S1: Graphical summary of the raw data.

Matrix of graphics representing the relationship of the factors *Cohort*, *Sex* (female, red; male, blue), beetle *Weight*, compound *Concentration*, and *Compound* to each other. For examples, (1) the *Weight* column x *Cohort* row graph shows the distribution of beetle weights for each sex, for each cohort; (2) the *Cohort* column x *Compound* row graph shows the number of beetles of each sex, for each cohort, and for each compound. In this graph one can see that equal numbers of beetles of each sex for each compound were tested, and that (-)-beta-phellandrene and (+)-beta-pinene were only tested with cohort 2.

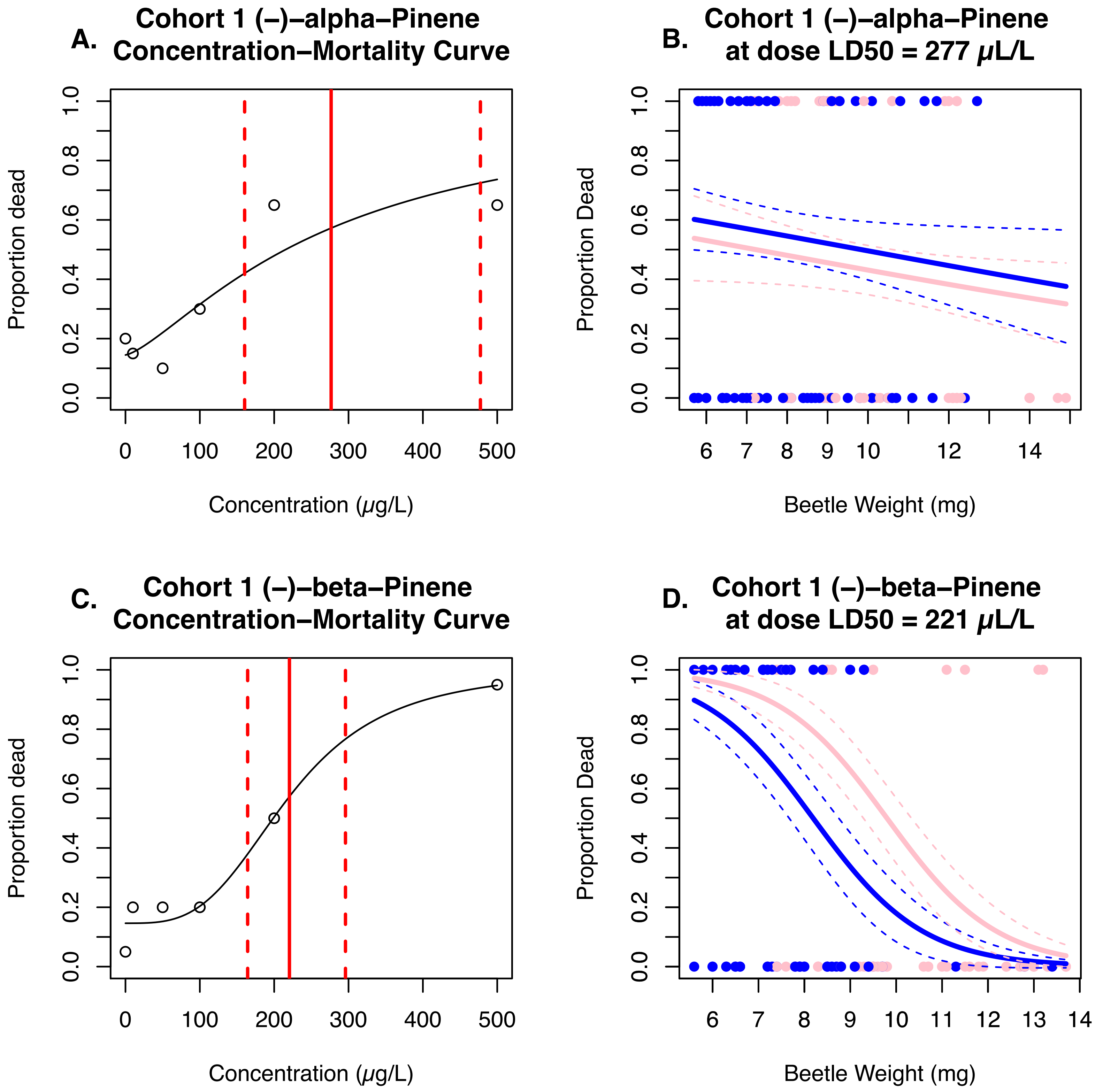

A.
Cohort 1 (−)−alpha−Pinene
Concentration−Mortality Curve
Proportion dead
Concentration (μg/L)
B.
Cohort 1 (−)−alpha−Pinene
at dose LD50 = 277 μL/L
Proportion Dead
Beetle Weight (mg)
C.
Cohort 1 (−)−beta−Pinene
Concentration−Mortality Curve
Proportion dead
Concentration (μg/L)
D.
Cohort 1 (−)−beta−Pinene
at dose LD50 = 221 μL/L
Proportion Dead
Beetle Weight (mg)

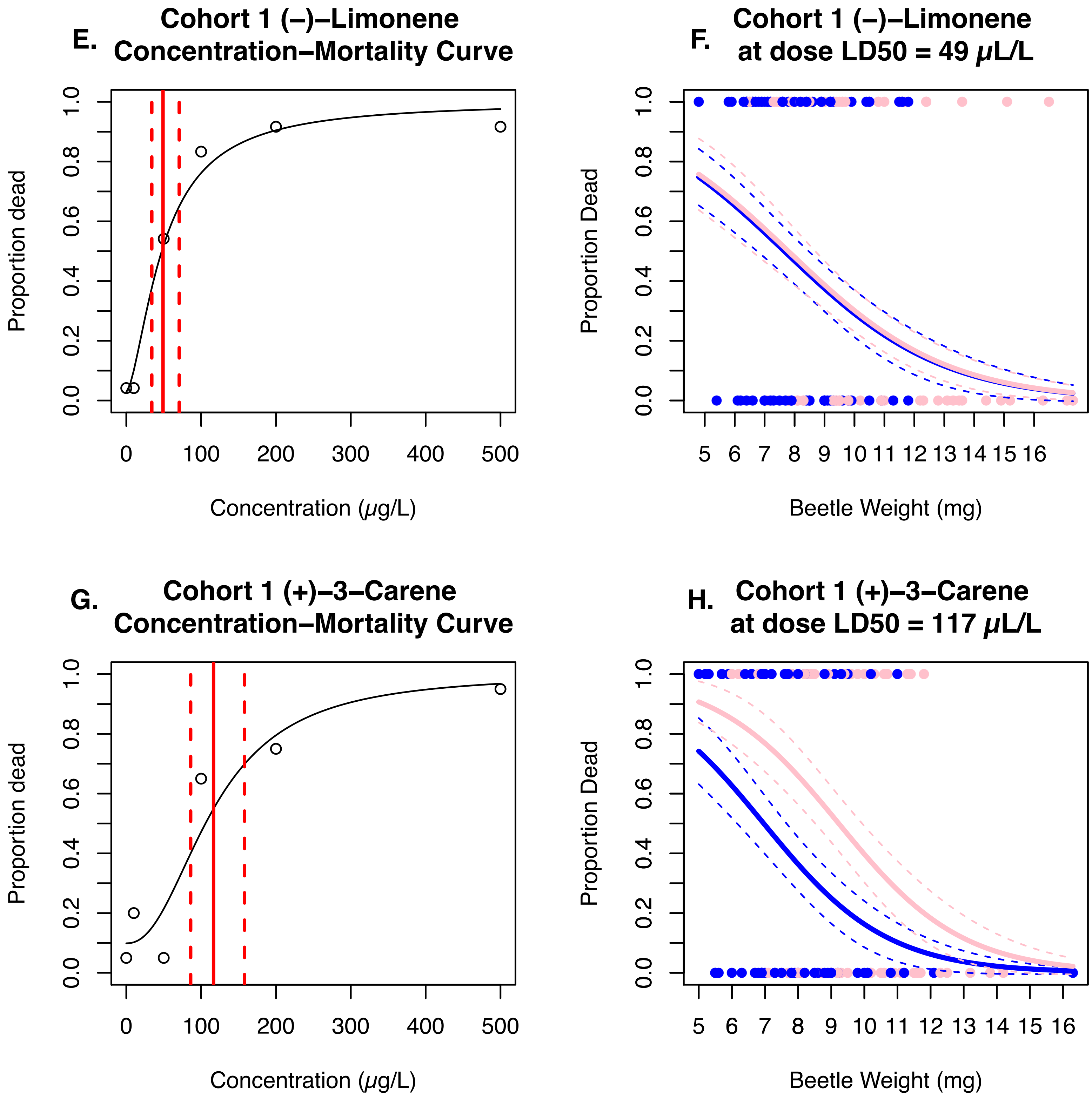
E.
Cohort 1 (–)–Limonene
Concentration–Mortality Curve
Proportion dead
0.0 0.2 0.4 0.6 0.8 1.0
0 100 200 300 400 500
Concentration (μg/L)
F.
Cohort 1 (–)–Limonene
at dose LD50 = 49 μL/L
Proportion Dead
0.0 0.2 0.4 0.6 0.8 1.0
5 6 7 8 9 10 11 12 13 14 15 16
Beetle Weight (mg)
G.
Cohort 1 (+)–3–Carene
Concentration–Mortality Curve
Proportion dead
0.0 0.2 0.4 0.6 0.8 1.0
0 100 200 300 400 500
Concentration (μg/L)
H.
Cohort 1 (+)–3–Carene
at dose LD50 = 117 μL/L
Proportion Dead
0.0 0.2 0.4 0.6 0.8 1.0
5 6 7 8 9 10 11 12 13 14 15 16
Beetle Weight (mg)

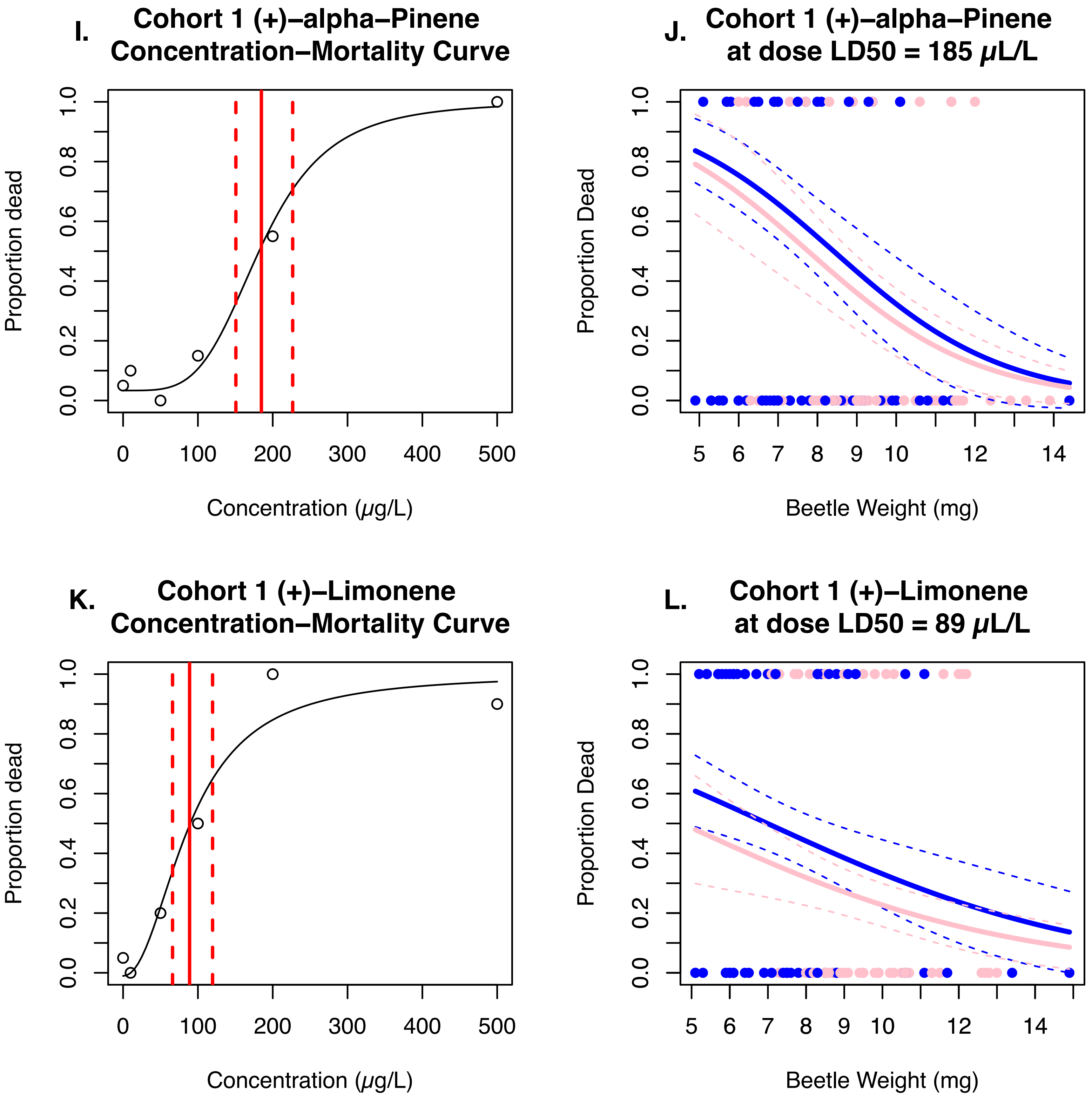
I.
Cohort 1 (+)–alpha–Pinene
Concentration–Mortality Curve
Proportion dead
0.0 0.2 0.4 0.6 0.8 1.0
0 100 200 300 400 500
Concentration (μg/L)
J.
Cohort 1 (+)–alpha–Pinene
at dose LD50 = 185 μL/L
Proportion Dead
0.0 0.2 0.4 0.6 0.8 1.0
5 6 7 8 9 10 12 14
Beetle Weight (mg)
K.
Cohort 1 (+)–Limonene
Concentration–Mortality Curve
Proportion dead
0.0 0.2 0.4 0.6 0.8 1.0
0 100 200 300 400 500
Concentration (μg/L)
L.
Cohort 1 (+)–Limonene
at dose LD50 = 89 μL/L
Proportion Dead
0.0 0.2 0.4 0.6 0.8 1.0
5 6 7 8 9 10 12 14
Beetle Weight (mg)

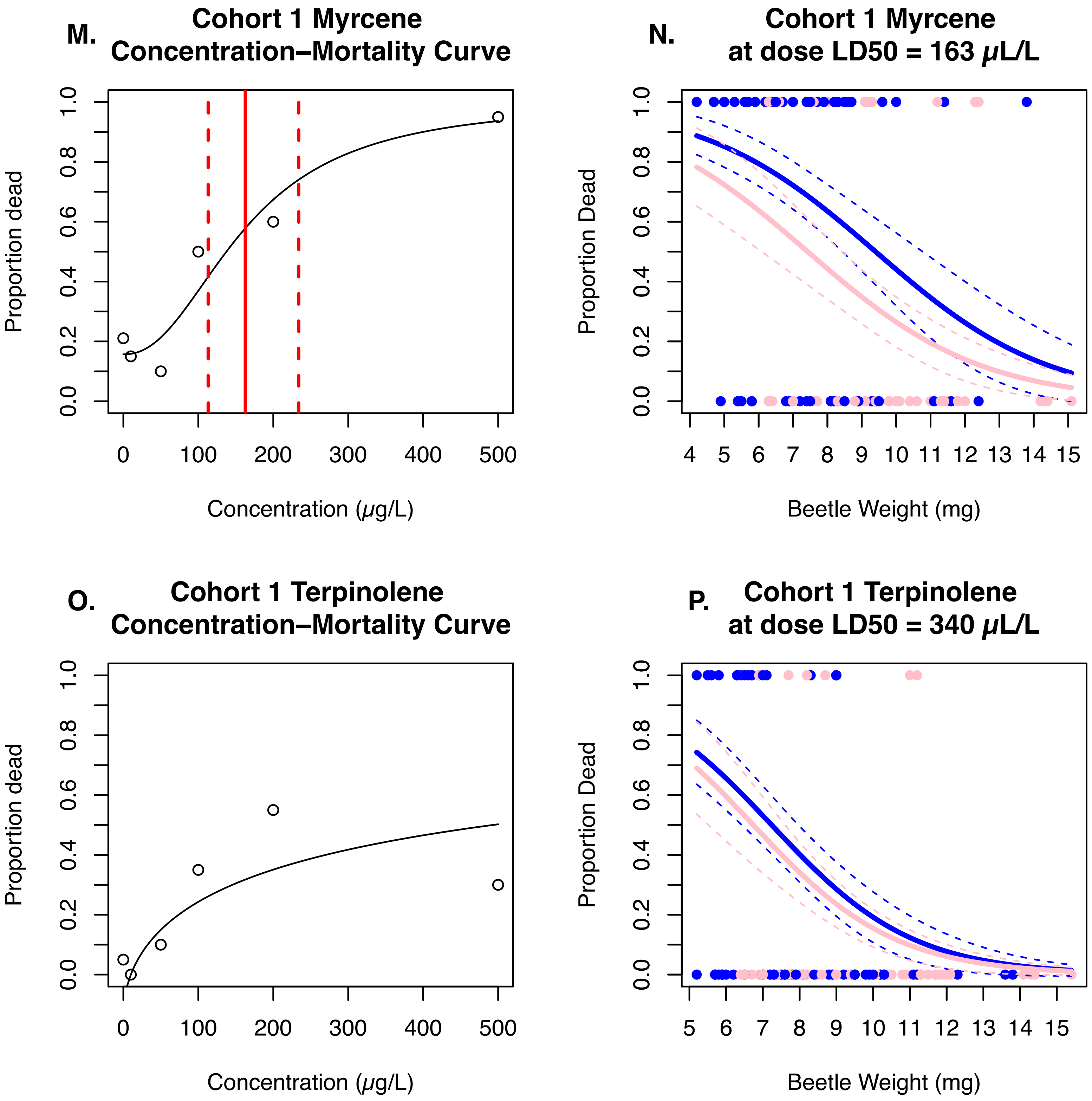
M.
Cohort 1 Myrcene
Concentration–Mortality Curve
Proportion dead
0.0
0.2
0.4
0.6
0.8
1.0
0
100
200
300
400
500
Concentration (μg/L)
N.
Cohort 1 Myrcene
at dose LD50 = 163 μL/L
Proportion Dead
0.0
0.2
0.4
0.6
0.8
1.0
4
5
6
7
8
9
10
11
12
13
14
15
Beetle Weight (mg)
O.
Cohort 1 Terpinolene
Concentration–Mortality Curve
Proportion dead
0.0
0.2
0.4
0.6
0.8
1.0
0
100
200
300
400
500
Concentration (μg/L)
P.
Cohort 1 Terpinolene
at dose LD50 = 340 μL/L
Proportion Dead
0.0
0.2
0.4
0.6
0.8
1.0
5
6
7
8
9
10
11
12
13
14
15
Beetle Weight (mg)

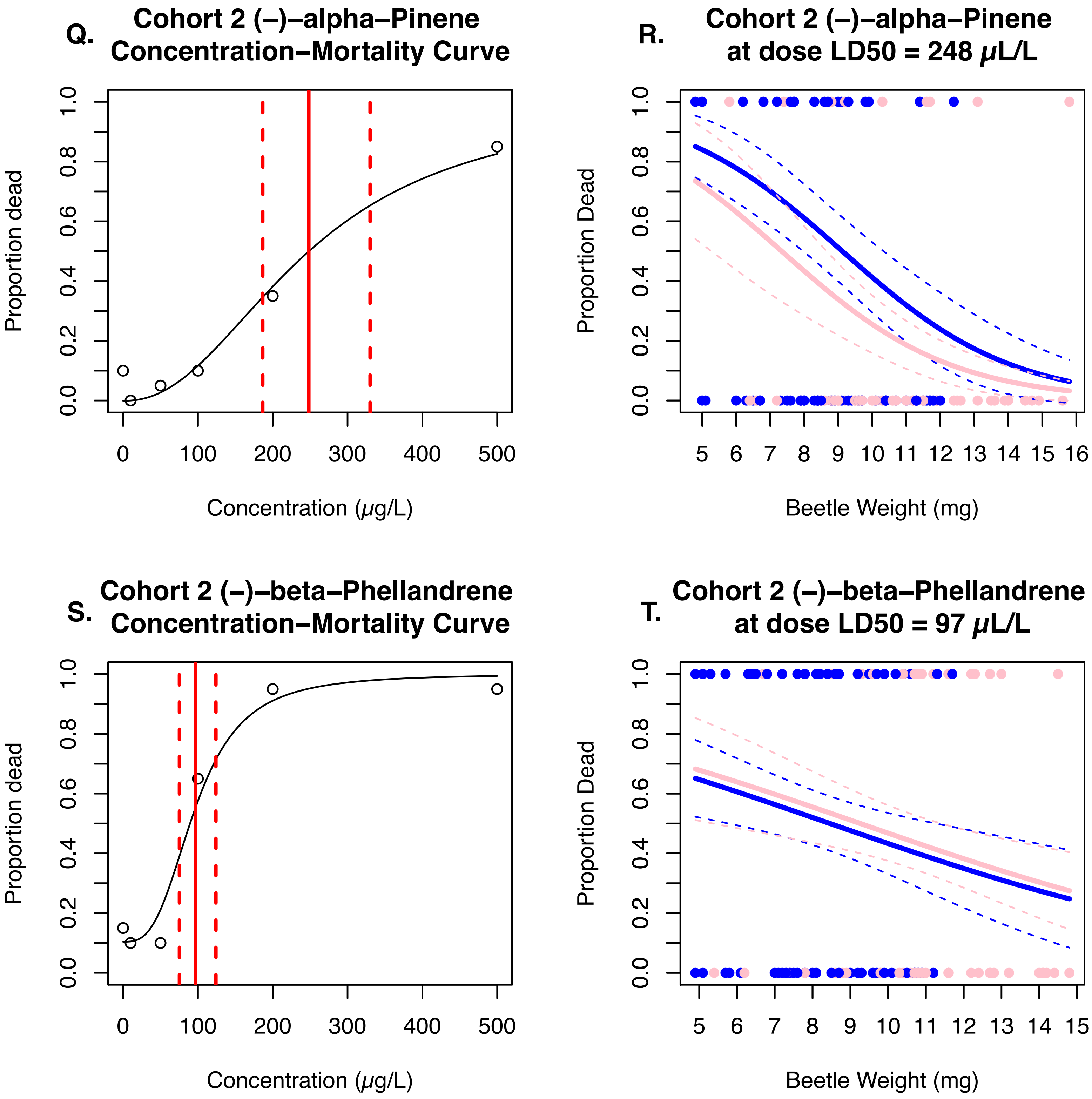

Q.
Cohort 2 (–)–alpha–Pinene
Concentration–Mortality Curve
Proportion dead
Concentration (μg/L)
R.
Cohort 2 (–)–alpha–Pinene
at dose LD50 = 248 μL/L
Proportion Dead
Beetle Weight (mg)
S.
Cohort 2 (–)–beta–Phellandrene
Concentration–Mortality Curve
Proportion dead
Concentration (μg/L)
T.
Cohort 2 (–)–beta–Phellandrene
at dose LD50 = 97 μL/L
Proportion Dead
Beetle Weight (mg)

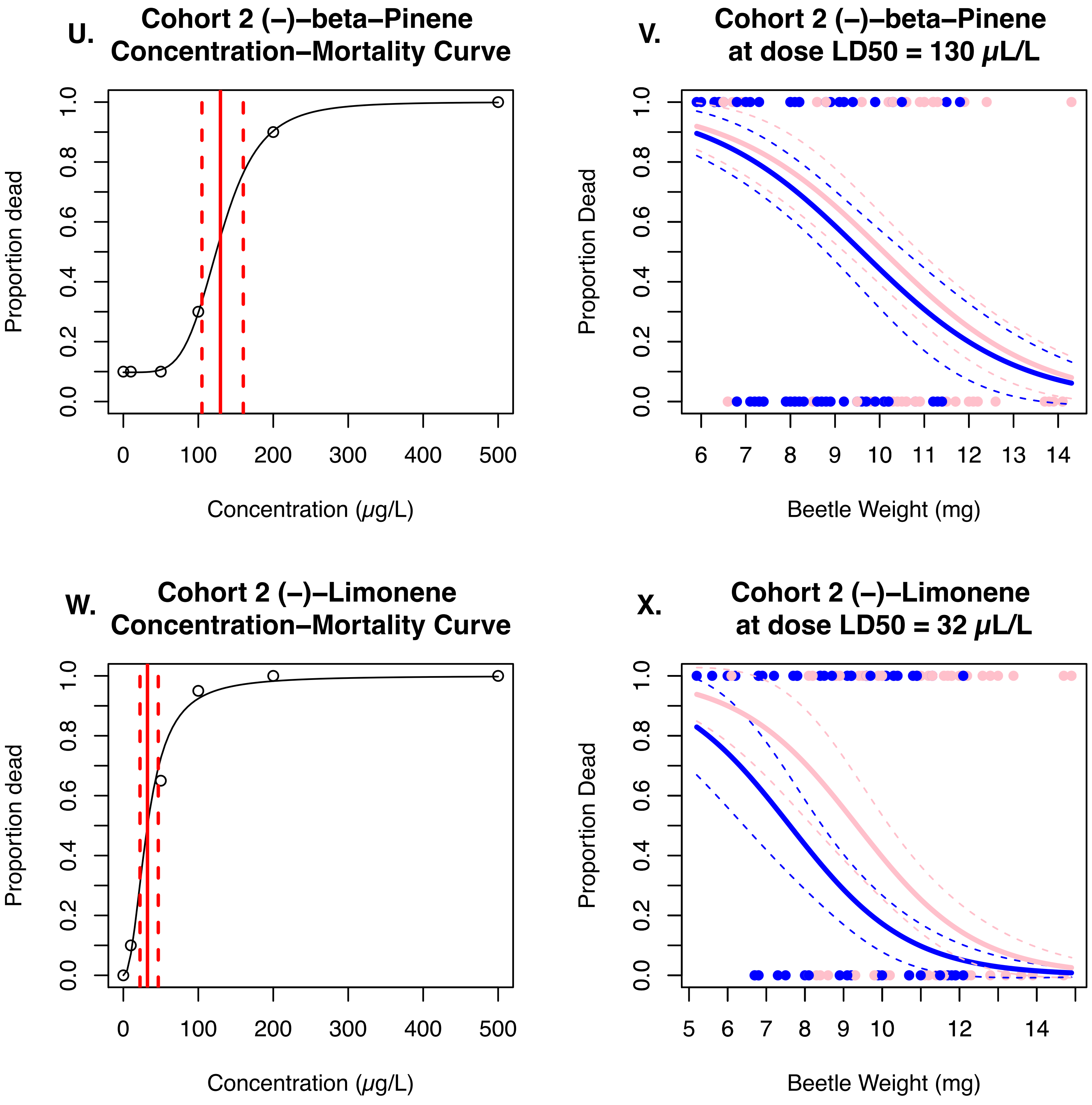
U.
Cohort 2 (−)−beta−Pinene
Concentration−Mortality Curve
Proportion dead
Concentration (μg/L)
V.
Cohort 2 (−)−beta−Pinene
at dose LD50 = 130 μL/L
Proportion Dead
Beetle Weight (mg)
W.
Cohort 2 (−)−Limonene
Concentration−Mortality Curve
Proportion dead
Concentration (μg/L)
X.
Cohort 2 (−)−Limonene
at dose LD50 = 32 μL/L
Proportion Dead
Beetle Weight (mg)

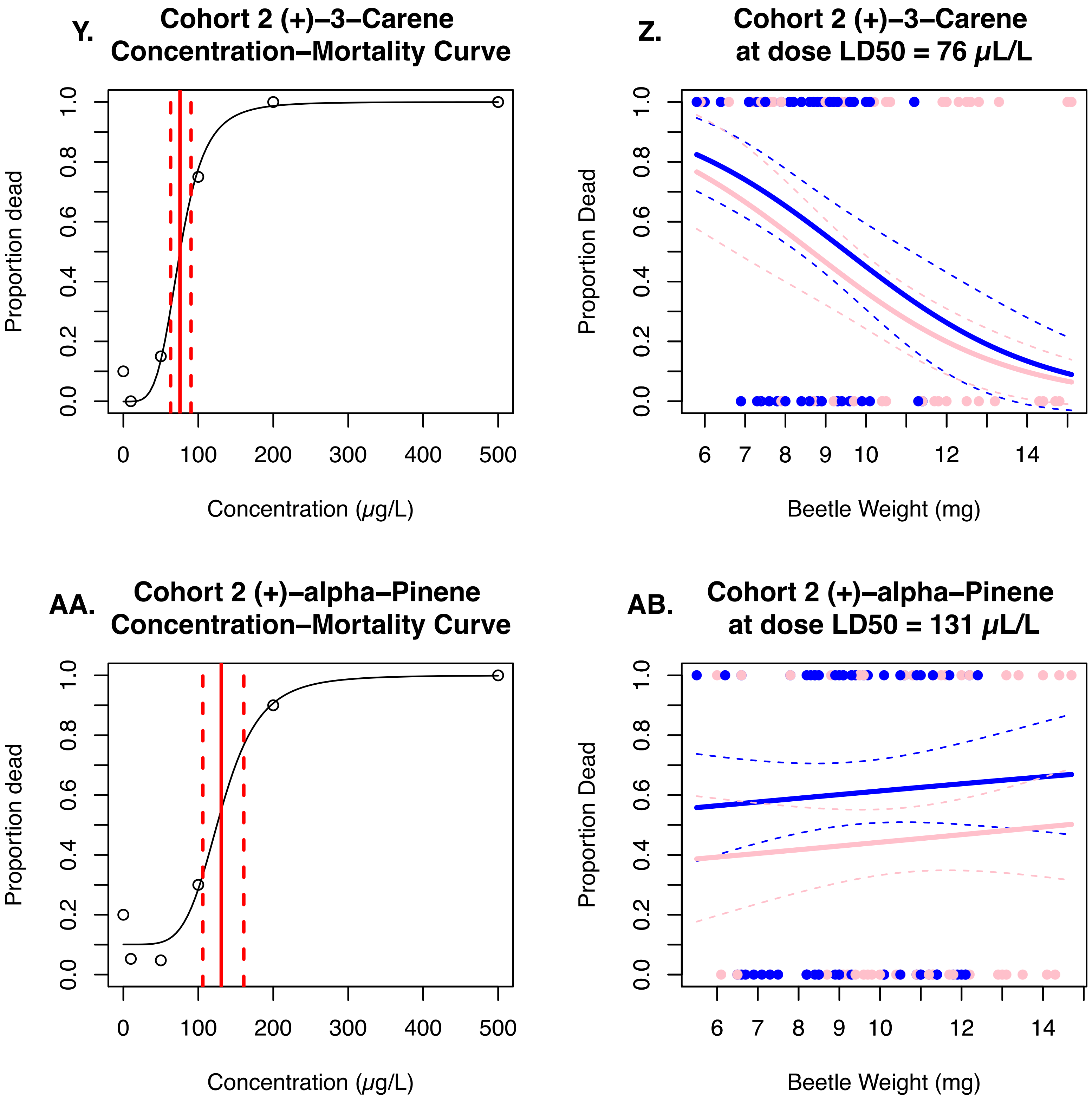
Y.
Cohort 2 (+)–3–Carene
Concentration–Mortality Curve
Proportion dead
Concentration (μg/L)
Z.
Cohort 2 (+)–3–Carene
at dose LD50 = 76 μL/L
Proportion Dead
Beetle Weight (mg)
AA.
Cohort 2 (+)–alpha–Pinene
Concentration–Mortality Curve
Proportion dead
Concentration (μg/L)
AB.
Cohort 2 (+)–alpha–Pinene
at dose LD50 = 131 μL/L
Proportion Dead
Beetle Weight (mg)

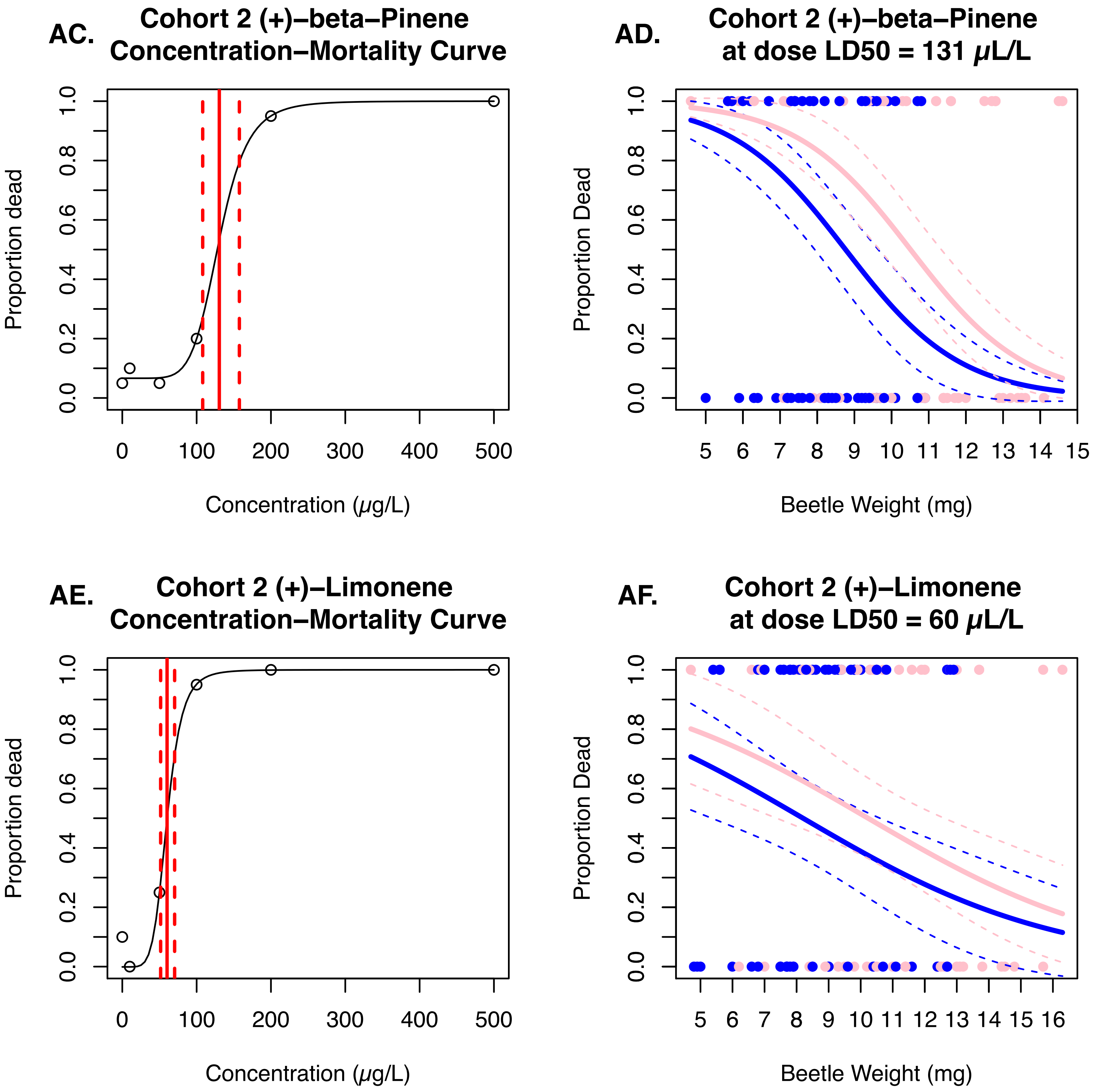
AC.
Cohort 2 (+)–beta–Pinene
Concentration–Mortality Curve
Proportion dead
Concentration (μg/L)
AD.
Cohort 2 (+)–beta–Pinene
at dose LD50 = 131 μL/L
Proportion Dead
Beetle Weight (mg)
AE.
Cohort 2 (+)–Limonene
Concentration–Mortality Curve
Proportion dead
Concentration (μg/L)
AF.
Cohort 2 (+)–Limonene
at dose LD50 = 60 μL/L
Proportion Dead
Beetle Weight (mg)

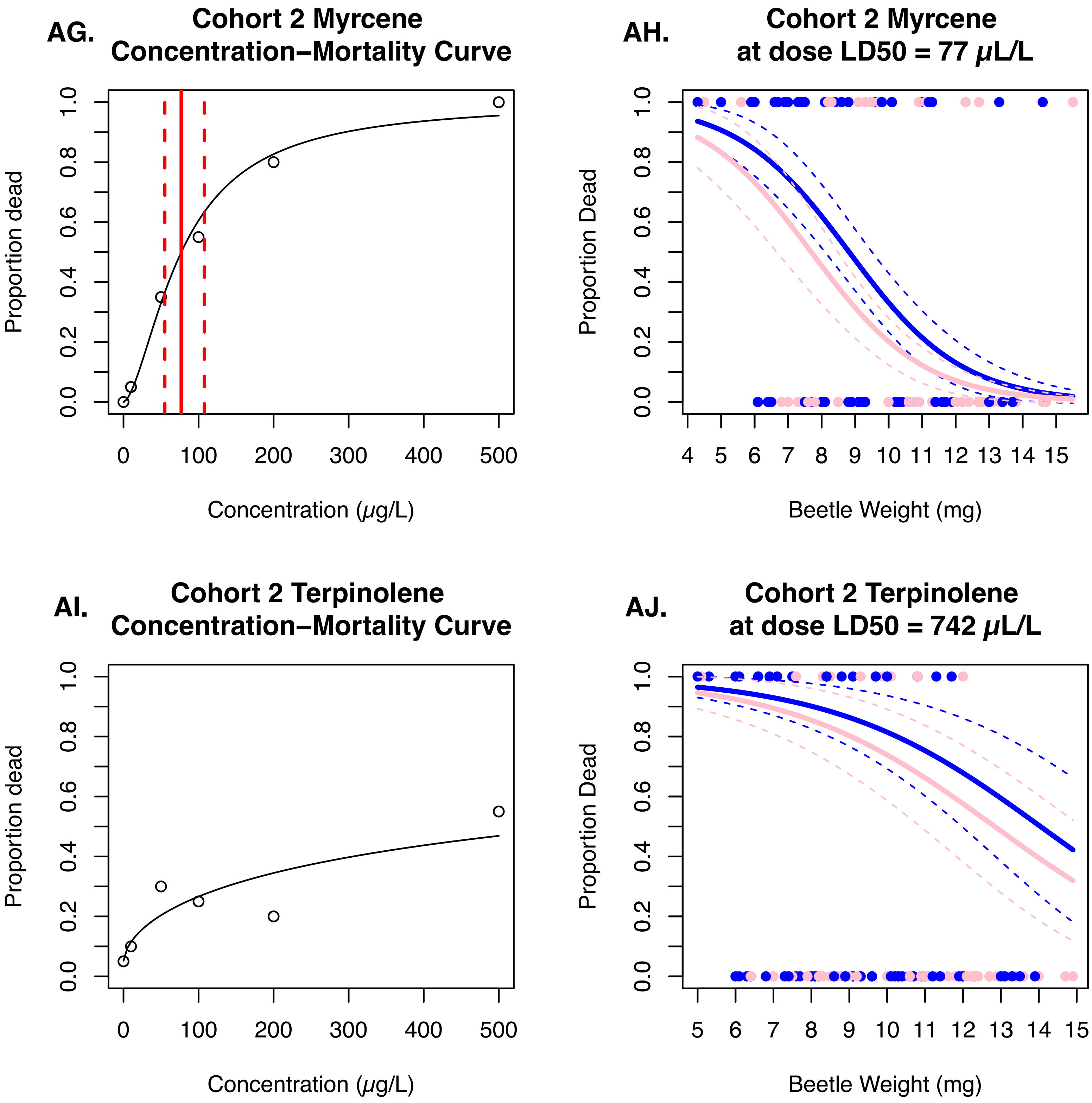
AG.
Cohort 2 Myrcene
Concentration–Mortality Curve
Proportion dead
Concentration (μg/L)
AH.
Cohort 2 Myrcene
at dose LD50 = 77 μL/L
Proportion Dead
Beetle Weight (mg)
AI.
Cohort 2 Terpinolene
Concentration–Mortality Curve
Proportion dead
Concentration (μg/L)
AJ.
Cohort 2 Terpinolene
at dose LD50 = 742 μL/L
Proportion Dead
Beetle Weight (mg)

Figure S2: LC_{50} and logit curves for each compound and cohort.

Subfigures A, C, E, G, I, K, M, O, Q, S, U, W, Y, AA, AC, AE, AG, and AI: Concentration-mortality curves for each compound and cohort. Dots represent the proportion of beetles dead at each concentration tested. The solid red line red indicates the LC_{50} value. Dotted red lines indicate the fiducial limits of the LC_{50} value.

Subfigures B, D, F, H, J, L, N, P, R, T, V, X, Z, AB, AD, AF, AH, and AJ: The predicted relationship between beetle weight and mortality for each sex at the LC_{50} value. The solid lines represent the predicted relationship between beetle weight and mortality at the LC_{50} based on logistic regression. Lines that slope down towards the right indicate that heavier beetles survive more often. The further apart the male (blue) and female (pink) lines are, the greater the difference in mortality between the sexes, independent of weight. Dotted lines indicate the 95% confidence intervals. The dots represent each beetle tested that either survived (y=0) or died (y=1) after treatment at all doses.

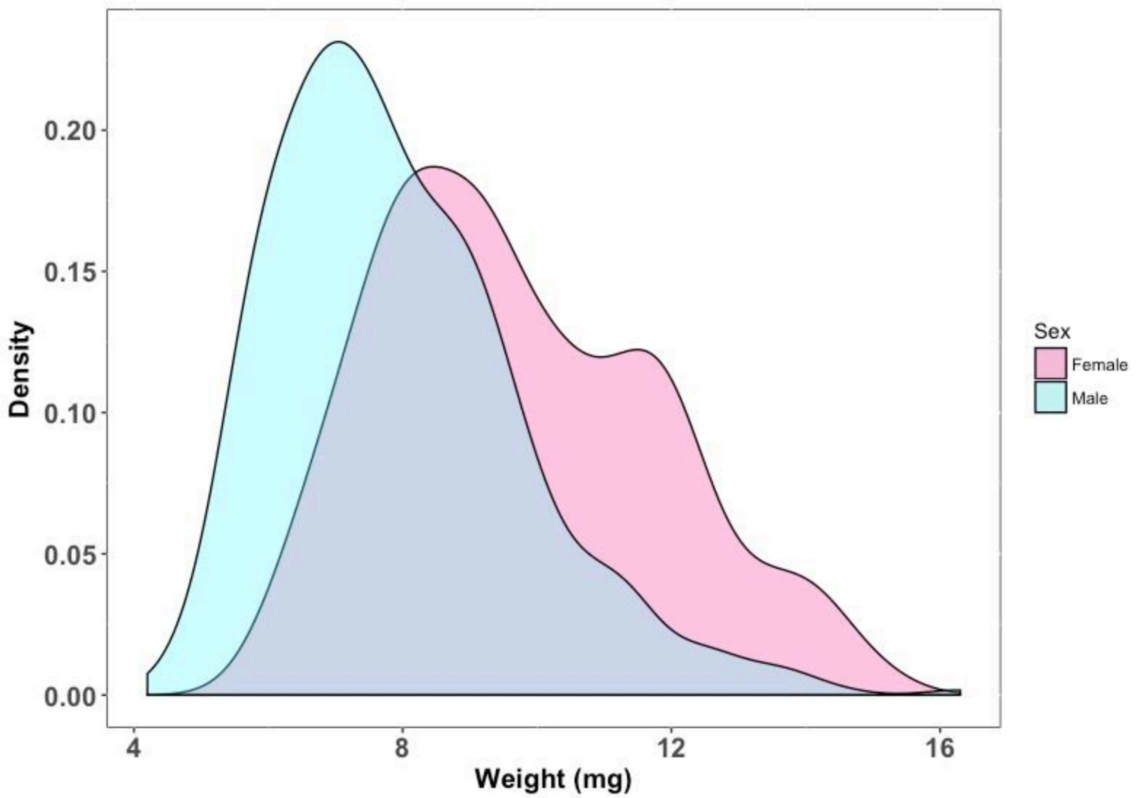

Fig. S3 Weight distribution of MPB from cohort 1

Average weight (±SD) = 8.81 ± 2.24 mg

Female average weight (±SD) = 9.73 ± 2.20 mg (N=492)

Male average weight (±SD) = 7.90 ± 1.87 mg (N=491)

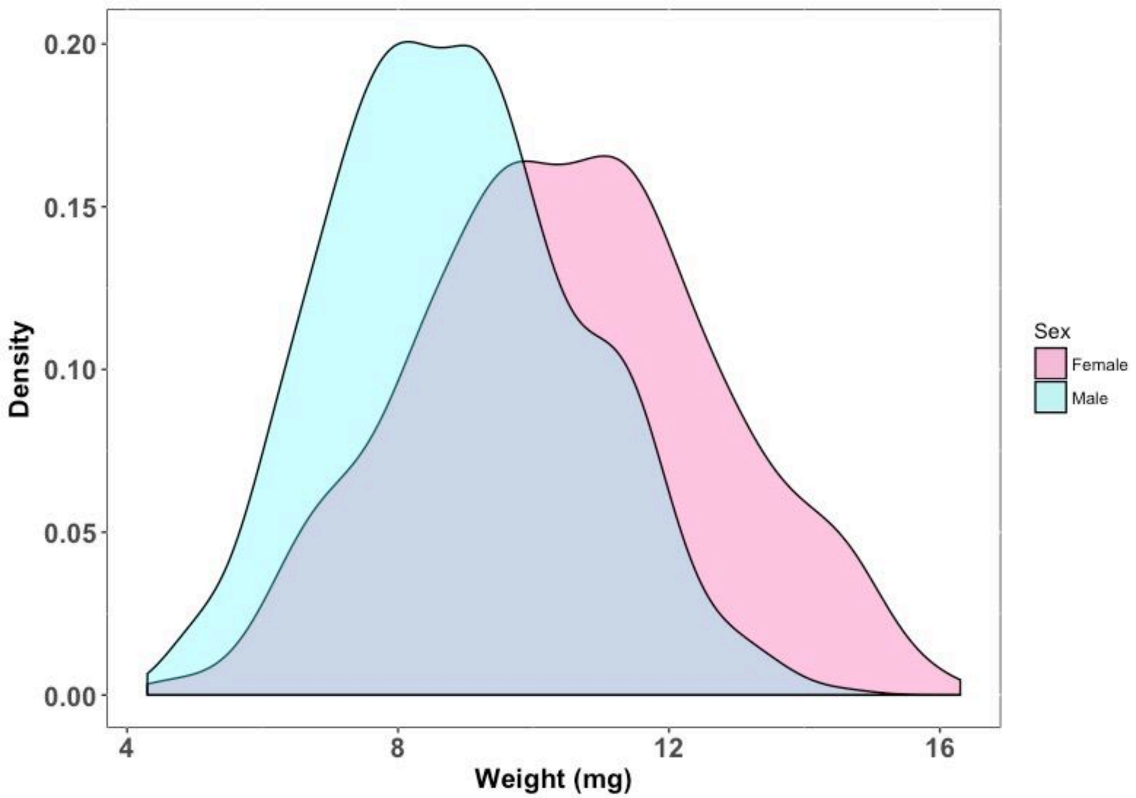

Fig. S4 Weight distribution of MPB from cohort 2

Average weight (±SD) = 9.69 ± 2.24 mg

Female average weight (±SD) = 10.53 ± 2.24 mg (N=599)

Male average weight (±SD) = 8.85 ± 1.90 mg (N=600)
